# Cows that are less active in the chute have more optimal grazing distribution

**DOI:** 10.1038/s41598-024-84090-z

**Published:** 2025-01-02

**Authors:** Maggie Creamer, Kristina Horback

**Affiliations:** Department of Animal Science, University of California, Davis, 1 Shields Avenue, Davis, CA 95616 USA

**Keywords:** Cattle, Behavior, GPS-tracking collars, Rangeland, Sustainability, Consistent individual differences, Environmental impact, Animal behaviour, Sustainability

## Abstract

Individual grazing patterns among cattle can contribute to sustainability of land use, however, little is known about the consistency of these grazing patterns. To address this knowledge gap, fifty Angus x Hereford cows were observed in repeated assays: A management assay (handling procedure, narrow chute, hydraulic squeeze), a social-feed trade-off assay (SFTA; choice between social mates and feed item), and novel approach assay (choice between social mates and feed item with novel pattern). The same cattle were tracked with GPS collars over two grazing seasons (June-August 2021 and 2022) and average grazing-related metrics (e.g., elevation used, distance traveled) were calculated within each season. Cows with a more passive response in the chute were found at higher elevation (*p* = 0.017), further from water (*p* = 0.043), and closer to supplement sites (*p* = 0.029). Cows that had higher latency to supplement in the SFTA traveled shorter distances on rangeland (*p* = 0.035). Thus, there was some evidence that cows with a more passive response to isolation and management had more optimal grazing patterns (grazed underutilized areas of the range at higher elevations and further from water sources). Selecting cattle with more optimal foraging patterns based on observable behaviors during handling and isolation could improve the sustainability of rangeland grazing.

**Supplementary Information:**

The online version contains supplementary material available at 10.1038/s41598-024-84090-z.

Consistent individual differences in behavior (CIDs) refer to relatively stable, behavioral traits in animals^1–3^ where some behaviors may be consistent across time, yet are context-dependent, while others span multiple contexts. Testing behaviors while animals are isolated *versus* in a social context is especially important for gregarious herding or flocking specie which are highly influenced by the behavior and presence of their conspecifics^4–6^. It is an open question of how and whether behaviors exhibited in isolation predict behaviors in social contexts [^7^, empirical example: ^8^]. Measuring cattle behavior in the same tests repeated over time is crucial to determining temporal consistency of behavior. Consistency of behavior (temporal or contextual) is primarily evaluated by determining repeatability, a ratio of among individual differences over among and within individual differences^9^. Cattle (*Bos taurus*) are a herding species which have been reported to display consistency of behavior in repeated temperament and personality assessments in mildly stressful environments and in isolation^10–12^. Determining whether these CIDs are only exhibited in specific contexts (i.e. in isolation) or are indicative of an underlying trait of the animal that is exhibited in other contexts and related to outcome measures may help to inform selective breeding practices or herd selection regimes^10^.

Temperament of cattle, often described as docility or excitability when humans are present or when cattle are handled, has been shown to relate to a variety of behaviors across contexts, such as maternal behavior^13^, breeding^14^, feeding behavior and weight gain^15–17^, social behavior^15^, and activity^18,19^. There are multiple reports in the literature that higher-arousal or more excitable cattle have lower feed intake and/or weight gain than their counterparts^20–22^. Cows and sheep which show a more active response to isolation, restraint, and human interaction have been shown to be more socially cohesive, or more proximate to conspecifics, than less responsive, or calmer, individuals^23–26^. CIDs, noted previously, and seen in the above examples in the literature, are found to relate across time and context, meaning that sets of behaviors seen in isolation may be related to an entirely different context, such as grazing behavior.

Beef cattle in California graze expansive landscapes called rangelands, that host a variety of ecosystem services (e.g. pollination, carbon sequestration, maintenance of native vegetation and habitats;^27^). The ways in which cattle graze have implications for the sustainability and conservation of these rangelands and the ecosystem benefits they provide^28,29^. Two studies on rangeland beef cattle by Wesley et al.^30^ (and expanded upon by Goodman et al.^31^), , and Wyffels et al.^32^ examine grazing patterns on rangeland through the framework of behavioral syndromes. They found contradictory results that a faster supplement consumption rate did^30^ and did not^32^ relate to more optimal, expansive grazing behavior. Bailey et al.^33^ found no relationship between temperament (i.e. aggressiveness toward calf handlers) at calving (a scale of 1 to 6) and grazing distribution of rangeland beef cattle. Neave et al.^34^ found that more calm and investigative dairy cattle had greater grazing time on 0.75 ha pastures, which is a small fraction of the typical hectarage grazed by beef cattle (upwards of 4,000 hectares on rangeland pastures according to data compiled by the USDA, Census of Agriculture^35^). To our knowledge, no studies to date have used comprehensive behavior assessments (i.e. multiple behaviors recorded across different situations) in beef cattle and related the results of these assessments to grazing behavior on rangeland.

Due to the fact that CIDs in cattle have been shown to relate to other outcome measures described above, and because rangeland beef cattle are often inaccessible by grazing such large expanses of land, it is important to consider best ways of measuring CIDs in practical, management settings. Current selection and breeding regimes are propelled by reproductive and other production-related traits^36^, however increasingly more literature is focused on understanding the interplay of behavior with these traits and other health outcomes for selection and breeding^10^. To actually use CIDs in behavior to predict these, and other emergent, outcomes (i.e. to use observation of cattle to one’s advantage in selection and breeding), ranchers must be able to observe meaningful behaviors directly and within their workflow. Many working ranches already have the infrastructure to isolate cows (for health checks, transportation, etc.), using these in practical behavior assays is paramount to translating cattle behavior research to working ranches.

The aim of this study was to determine whether behaviors from experimental behavior assays, that are practical to implement on most ranches, were related to grazing patterns on rangeland. Cattle underwent three experimental behavior essays (chute handling, social-feed trade-off and novel item;^37^) and were GPS-tracked while grazing rangelands^38^. Drawing parallels from the literature highlighted above in this introduction regarding how calm cattle consume more feed and more active cattle are found more proximal to groupmates, as well as how cattle that feed more from supplement graze more expansive areas, we have formulated the following hypotheses and predictions. If behaviors from the management assay and feeding behavior from the preference assays predict rangeland use metrics, we would expect that cattle that were (a) calmer in the management assay while handled and during isolation and (b) approached the supplement more quickly in both the social-feed trade-off and novel approach assays will be those that travel farther, higher, cover more area to graze on rangeland, and have lower degree strength (i.e. less strong associations with other cattle) in the social network than cattle that are more active or excitable and/or that did not approach supplement quickly.

## Methods

### Animals and housing

Fifty Angus x Hereford cows were selected for this study if they were at least parity one heifers and were within the range of 80–100 days pregnant determined via ultrasound. Study cows ranged from 2 to 8 years old (mea*n =* 4.96, sd = 1.95), weighed between 457 and 857 kg (mea*n =* 608.06, sd = 69.93) and resided at the Sierra Foothill Research and Extension Center (SFREC) in Browns Valley, CA that is managed by University of California Agricultural and Natural Resources (UCANR). This cattle herd represents the typical age and breed composition of rangeland cattle herds in the Sierra Nevada foothills region. We administered three different repeated behavior assays across two years: (1) observations of behavior while cattle were handled into and traversed a chute (management assay), (2) a preference assay that featured a social-feed trade-off, and (3) a preference assay that featured a novel item approach^37^. The same cattle were tracked with GPS collars while they grazed on rangeland across two summers^38^ and various grazing-related metrics were obtained from these data including home range area and social network information.

### GPS tracking and pasture

All fifty cows were collared with custom-built GPS collars (Knight Collars^39^), , which were leather collars with a Polyvinyl Chloride (PVC) box that contained a GT-120 iGotU GPS tracker (Mobile Action, Taiwan), and a rechargeable Li-ion battery pack. The units were programmed to record location every 10 min. According to Morris and Conner^40^, these particular GT-120 iGotU trackers have a location error of less than 10 m, a mean 50% circular error probable of less than 7 m, and fix success rate (i.e. the rate at which the GPS unit can successfully communicate with the satellite to record location) is not significantly affected by cover.

Study cattle grazed a fenced 2.53 sq. kilometer (253 hectare), oak woodland rangeland pasture across two years (2021 and 2022) in months June-August. Cows were familiar with the climate and environment of SFREC and grazed at a stocking density of 0.64 AUM per hectare (according to UC Rangelands Animal Unit Calculator^41^), which as a low stocking rate is typical on California extensive rangeland.

To collect minimum, maximum, and accurate average daily temperatures in the pasture, Onset HOBO data loggers™ were placed in a solar radiation shield and set to collect temperature data every 30 min. In 2021, the average minimum temperature during the grazing period was 21.7 °C, the average maximum daily temperature was 39.4 °C, and the average mean daily recorded temperature was 28.9 °C. In 2022, the minimum and average mean temperatures were slightly lower. The average minimum daily temperature was 20 °C, the average maximum daily temperature was 39.4 °C, and the average mean daily temperature was 27.8 °C.

**Fig. 1:**
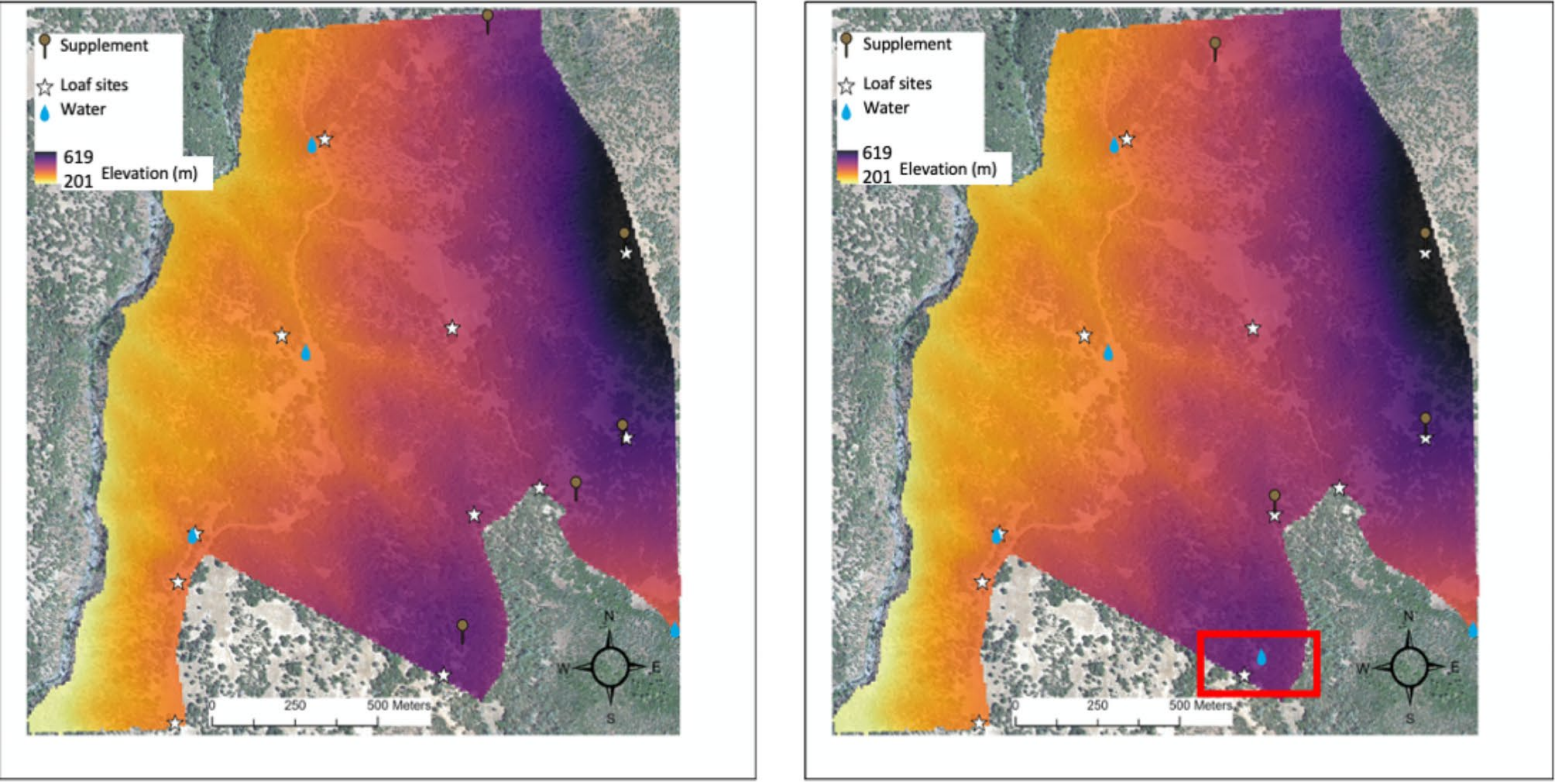
This figure shows the grazing pasture characteristics. Elevation is represented by the color scale (legend provided) and the accurate boundary and size (in meters) is also shown with the scale bar. Loaf sites, water sites, and supplement sites are indicated with symbols where they are placed in the pasture. The placement of sites in year 1 (2021) is displayed on the left and year 2 (2022) is on the right. A new pipe-fed water trough was added between the 2021 and 2022 grazing seasons and its location is labeled by the red box on the right side of the figure (reproduced from Creamer & Horback^38^).

A more detailed description of the study pasture is described in Creamer and Horback^38^. About 19% of this pasture was classified as open grassland *versus* 81% tree cover. Elevation in this pasture ranged from 201 to 618 m (Fig. 1) and slope ranged from 0 to 47 degrees, with an average slope of 16 degrees. Lower elevation areas of the pasture and a few areas towards the top of the pasture were fairly steep and rocky. Vegetation was diverse with representation from both native rangeland species and invasive rangeland species, and also palatable (e.g. wild oats and wheat grasses) and non-palatable (e.g. star thistle) species for cattle.

Loafing sites were identified by ranch managers and by observation in the first 4 weeks of data collection, and eleven of these sites were recorded for use in data analysis. Supplement sites were chosen by ranch managers to encourage grazing at higher elevation and on rugged terrain and were kept relatively consistent across years. The water sites were fixed pipe-fed troughs that had been established in the pasture for easy cattle access. A new water site, another pipe-fed trough, was added in year two at a higher elevation. Figure 1 shows study pasture characteristics including where loafing sites, supplement sites (both mineral supplement and low moisture protein blocks), and water sites can be found between both years.

### Behavior and preference assays

**Fig. 2:**
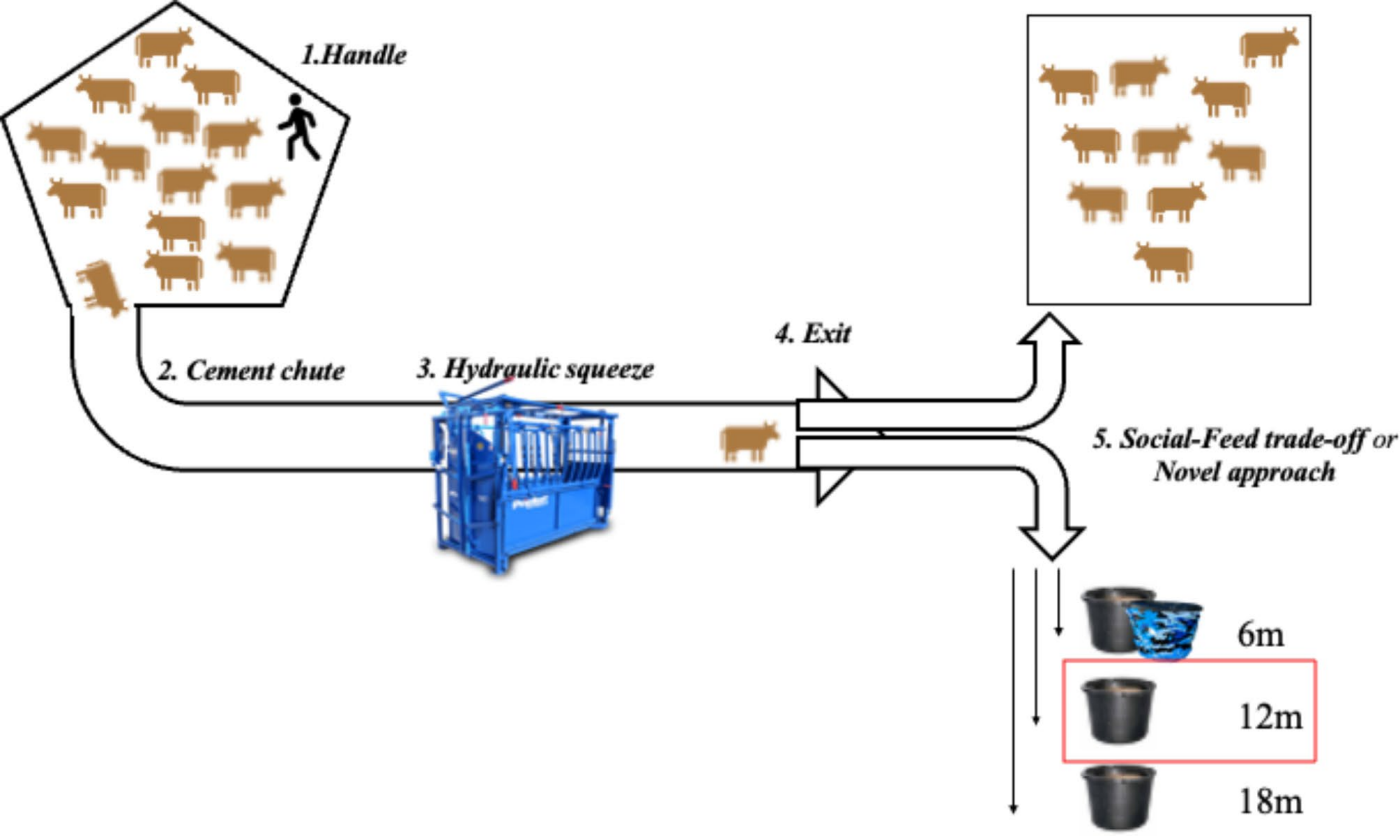
A diagram of the testing path for the management assay (1. Handle, 2. Cement chute, 3. Hydraulic squeeze, 4. Exit squeeze) followed by the 5. Social-Feed trade-off (with bucket being either 6, 12–18 m away) or the novel approach assay (with unfamiliar bucket being 6 m away). The response to a bucket at the 12 m distance in the Social-Feed trade-off trials was highly consistent within and between the years, and thus, retained for further analysis with grazing-related metrics.

Cattle behavior measures were obtained from a series of assessments that were administered in May-June in years 2021 and 2022 (Fig. 2). To complete all testing of individuals in the morning before the daily temperature drastically increased (before 1100), we split cattle into four groups of 12 or 13 cows balanced by age and pregnancy status; groups were kept consistent between years. Behavior tests commenced between 0730 and 1100 at a corral and chute which included a hydraulic squeeze chute. We began conducting behavior assessments one week after fence-line weaning and in year two of the study, we reversed the order of testing for subgroups to mitigate timing and post-weaning confounds.

The management assay was comprised of two situations: Cattle were handled by an experienced, but previously unfamiliar handler (in the corral) and cattle were isolated in the chute (specifically in the cement chute, hydraulic squeeze, and exit; Fig. 2). To begin behavior assessments, two subgroups of cows were herded into the holding pen by an experienced, familiar, facility manager. The same familiar manager sorted the testing subgroup into the corral before the chute and other subgroup was randomly separated into a social group (*n =* 10 individuals) and a social buffer group (*n =* 2 or 3 individuals depending on the size of the subgroup being tested). At the beginning of testing, the study group (*n =* 12 or 13) and the social buffer group (*n =* 2 or 3; totaling *n =* 15) were placed in the corral that leads into the chute and hydraulic squeeze chute.

A different handler, previously unfamiliar to cows on the first day of assessment, but kept the same throughout all behavior assessments with all subgroups, herded cows into the chute one at a time. The handler, who also kept their appearance the same every day of testing, approached and moved cows following a standardized protocol^37^. After the cow was herded individually into the chute and separated from the corral by a gate, each cow walked through the cement chute at their own pace and awaited at the closed doors of the hydraulic squeeze chute for 30s (seconds). After 30s had passed, a research assistant opened the squeeze chute doors at the same speed for each cow. At their own pace, cattle walked through and exited the hydraulic squeeze chute to a spray-painted line marking 2.5 m from the exit doors of the squeeze chute. This management assay was repeated 4 times each year for 2 consecutive years.

Once cattle exited the squeeze chute and crossed the 2.5 m line, they participated in the preference assay (social-feed trade-off or novel approach) for a total of 5 min, which was set up as shown in Fig. 2. The ‘social group’ of ten conspecifics from the other (non-test) subgroup were corralled in the alleyway on one side of the focal cow and were contained within 30 m of the gate separating the focal cow from conspecifics (this distance was chosen because it is biologically relevant to herd cohesion according to Stephenson et al.^42^). The separating gate allowed sensory contact (visual, olfactory, auditory) between the focal cow and conspecifics, it was used for physical separation only. Cattle chose to approach conspecifics on one side or to move towards a familiar bucket filled with supplement (~ 16 kg) to the opposite side that was at varying distance away from conspecifics across days (Fig. 2;^37^). For the purpose of the current study, we only analyzed behaviors when the bucket distance was 12 m away because latency to supplement had the highest correlation coefficient across years for that distance, and thus deemed the most consistent assay^37^. The novel approach assay, which was conducted after a one-day rest from the last social-feed trade-off assay was set up in the same T-maze configuration as the social-feed trade-off assay, but the bucket was covered with an unfamiliar color and pattern that was novel to cows and the distance of the bucket was at 6 m (Fig. 2).

Each repetition of the management assay and the preference assays were recorded by camcorders (Sony FDR-X3000 4 K Action Cameras, Sony Corporation of America, New York, NY, USA) on tripods (4 m high) that captured cattle positions clearly from above and to the sides of the chute and T-maze. Behaviors were coded from video with all-occurrence continuous sampling of states and events^43^ by trained and reliable observers^37^ using The Observer XT software v.11 (Noldus Information Technology, Wageningen, The Netherlands).

We recorded six behavior measures from the management and trade-off assays to represent CIDs (definitions provided in Table 1). From the management assay, we recorded (1) total time needed to handle cattle into the chute, (2) duration in the cement chute, (3) duration in the hydraulic squeeze without restraint, and (4) duration to exit the squeeze to 2.5 m. From the trade-off tasks, (5) latency to familiar supplement while the bucket was at 12 m and (6) latency to the novel bucket during the novel approach task^37^. All behaviors recorded from videos for this analysis and definitions can be found in the behavioral ethogram (Table 1).

**Table 1:** Ethogram of behaviors that were continuously recorded using all-occurrence sampling (reproduced from Creamer & Horback^37^).

Behavior	Definition
Handle duration (s)	Duration of time from when handler is able to isolate the cow of interest (the cow that eventually goes through the chute) with one or two other herd mates (handler is between cow of interest and herd mates) to when cow’s hindquarters cross through cement chute gate.
Chute duration (s)	Duration of time from when hindquarters (base of tail) cross the cement chute gate to when hindquarters cross chute exit gate (9.2 m).
Squeeze duration (s)	Duration of time from when entire cow head (behind ears) crosses squeeze gate to when hindquarters (base of tail) cross squeeze exit gate (3.4 m).
Exit duration (s)	Duration of time from when cow hindquarters cross squeeze exit gate to when cow hindquarters cross orange exit line at 2.5 m.
Latency to supplement (s)	Duration of time from when hindquarters (base of tail) crosses exit line to when cow takes first bite of supplement.

### Data cleaning

GPS data were imported from collars directly into ArcPro GIS software (GIS software by ESRI™, Version 2.5.0, Redlands, CA) along with the exact rangeland boundary (fence line) obtained with Trimble GEO 7x handheld GNSS receiver. A methods study on iGotU 120 device error by Morris and Conner^40^ noted 27 m was the 95% circular error probable with dense cover, thus locations outside of 27 m of the pasture fence were deemed improbably correct and removed from the dataset. Outliers in the GPS data that indicated the cow was running at a speed greater than 3 m/s for the full fix interval (10 min) were removed based on biological evidence of cattle running speeds^21^. Outliers were checked before and after this cleaning process with the R package ctmm^44^, and removing these points was effective in eliminating all previously flagged outliers (see more details on this in Creamer & Horback^38^), . GPS points that had zero satellite fixes were also removed from the data to reduce potential error.

We used ArcPro GIS tools and movement packages in R^45^ to calculate rangeland metrics for data analysis. To calculate daily trajectories (distance traveled) by cattle from GPS points, we used the AdehabitatLT package^46^ in R^45^. We computed distances to water sites, supplement sites, and loafing sites using the Near tool provided in ArcPro GIS software (GIS software by ESRI™, Version 2.5.0, Redlands, CA). We used a Digital Elevation Model with 1/3 arc second resolution of the study area and the ArcPro Extract Values to Point tool (GIS software by ESRI™, Version 2.5.0, Redlands, CA) to append elevation and slope data to each GPS data point. Daily values of elevation, slope use, and distances to water sites, supplement sites, and loafing sites were averaged across the week to evaluate broader, weekly changes in daily grazing patterns and to enhance model fit across years by aggregating the data to week (similar to Michelangeli et al.^47^). Before aggregating data to week, we filtered data to contain only days where there were at least a third of the total possible fixes for the day (threshold of greater than 48 GPS points per day, removed 1.7% of data).

Two rangeland use metric variables of interest were calculated from the full three months of data within year of grazing season. These were the 50% (core range) autocorrelated kernel density estimate of home range and the weighted degree strength of each individual in the social network. The ctmm package^44^ in R, which uses the CTSD (continuous-time speed and distance) method of approximating speed and distance^48^, was used to calculate autocorrelated kernel density estimators for the area of each individual cow’s 50% ‘core range’. Weighted degree strength is a centrality measure that is commonly used in animal social networks to reflect the social status of individuals and their influence on one another^49–51^. Undirected, weighted, degree centrality based on GPS proximity data with other collared cattle was calculated via the density of interactions with the spatsoc package in R^52,53^ such that interaction was defined as two cattle within 30 m of each other in a window of 5 min. The 30 m threshold was chosen based on previous literature indicating this distance captures appropriate subgroups of cattle and has undergone ground-truthing by visual observations^42,54^.

Elevation, slope use, distance to water and distance traveled^55,56^, as well as use of supplement^57,58^, are all metrics that have been studied in previous work regarding cattle grazing behavior, thus we also used these outcome measures for relevance and comparison with past work. We also included distance to loafing sites and social network degree strength because we were interested in both social and resting behaviors of cattle and considered this as a unique way to understand cattle proximity to each other and to resting sites. Adjusted kernel density estimates are often used in wild animal behavior research to understand ranging area^59^, and 50% home range was used because this herd is bounded by fence line, and this (as opposed to 95% home range) would distinguish cattle that *regularly* used larger areas from those that did not.

### Datasets between years

**Fig. 3:**
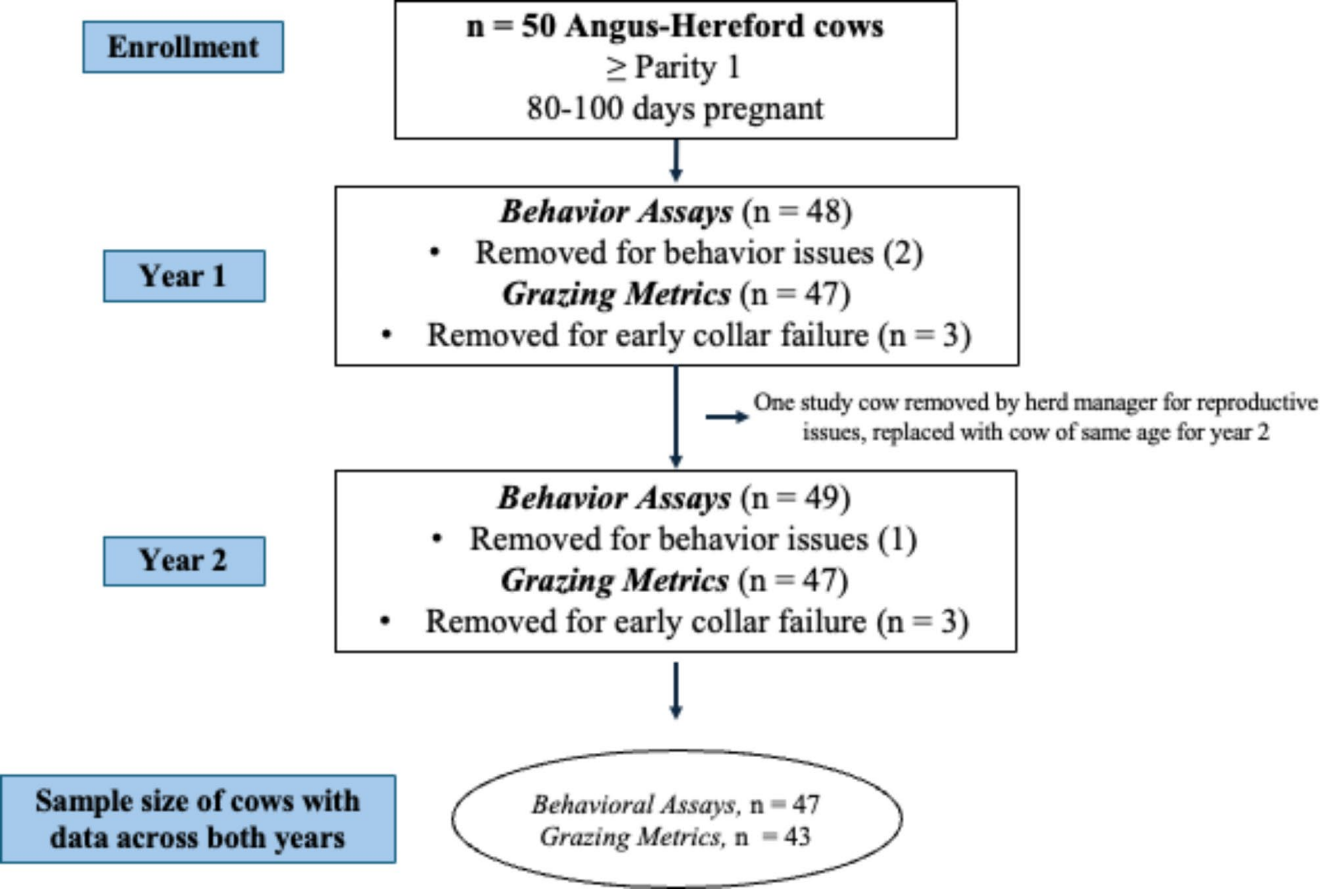
This infographic displays the sample sizes of cows across years and the reasons cows were excluded from either data collection or analysis.

In year one, 48 of the selected 50 study cattle participated in all behavior experiments and all 50 cows were tracked by GPS collars while grazing with 20 cohorts (non-study cows) on rangeland pasture for 70 days total (June 22, 2021 – Aug 30, 2021; Fig. 3), although some collars failed before the end of the grazing season (mean days tracked = 66.6, SD = 7 days). In year two (2022), 47 of the same 50 cows participated in behavior experiments; one cow was added in year two to replace a culled cow (due to concerning calving issues) after year one and one cow that had not completed all repetitions of behavior experiments in year one did complete all repetitions in year two (Fig. 3). In year two, 49 of the same 50 cattle were tracked with GPS collars and grazed with the same 20 non-study cows on rangeland pasture for 77 days total (June 15, 2022 – Aug 30, 2022; mean days tracked = 75.4, SD = 5.3 days). The 20 non-study cows remained the same in both years, they did not participate in behavior assays nor were tracked with GPS collars, but grazed on pasture with the herd to keep up with management standards of the appropriate AUM on the allotted rangeland pasture and to reflect typical ranch stocking densities (J. Munson, personal communication, February, 2021). Due to a few collars failing very early in the season or falling off, there were 47 cows with analyzable GPS datasets within each year, and 43 of the same cows with analyzable GPS datasets across both years (mean age year one = 4.98, SD = 1.94; Fig. 3).

### Ethical statement

All experimental protocols were approved by and carried out in accordance with the University of California Davis Institutional Animal Care and Use Committee (protocol #22672). This study is reported in accordance with ARRIVE guidelines^60^.

### Statistical analysis

#### Repeatabilities and correlations of assay-related metrics

Repeatabilities and correlations of behavior data were calculated in a previous study^37^, but here we are summarizing this process. We calculated repeatabilities of behaviors (see Table 1) from the management assay to ensure the first day of data could represent consistent individual variation in cattle behavior (i.e. a stable trait) in predictive models. We did not average behaviors for regression, as aggregation of repeated measures leads to information loss and can weaken important differences among individuals^61,62^. Repeatability of behavior is calculated by dividing among individual variance $\:{\sigma\:}_{\alpha\:}^{2}$ by the total variance, $\:{\sigma\:}_{\alpha\:}^{2}+\:{\sigma\:}_{\varepsilon }^{2}$^9^.

$$\frac{{\sigma _{\alpha }^{2}}}{{\sigma _{\alpha }^{2}+{\text{~}}\sigma _{\varepsilon }^{2}}}$$

We used the brms package in R^45,63^, an interface to Stan (Stan Development Team, 2023), to run Bayesian multilevel models to examine broad-sense repeatability^64^ of behaviors exhibited in repetitions of the management assay^37^. We included year as a predictor variable in these models to account for latent differences in behavior between years^64^ and used relatively weak, uninformative priors for these data. Repeatability, which has the same calculation as intra-class correlation coefficients for grouping variables, was calculated for cow ID using the variance_decomposition function in the performance package^65^. This function is the recommended way to estimate ICC or repeatability for Bayesian models because it uses the posterior predictive distribution and can be used with a wider variety of data types (i.e. ‘families’;^65^). Repeatabilities adjusted for year are reported in Table 2. Because repeatability must inherently be greater than or equal to zero (it is a ratio), if the lower bound of the confidence interval of the repeatability estimate was not close to zero (> 0.10), then the behavior was considered to be repeatable. Between-year correlations of latency to supplement in the familiar social-feed trade-off and novel approach were calculated with Spearman’s rank correlations. The Spearman’s rank correlation coefficient between years of the latency to supplement in the familiar (12 m distance) and novel assays are presented also in Table 2.

**Table 2:** This table displays both repeatability estimates of behaviors across repetitions of the management assay with 95% confidence intervals (CI), and Spearman’s rank correlations with p values of latencies in the social-feed trade-off assay and novel approach assay between years. Repeatability estimates were adjusted for year and calculated from bayesian mixed models. We used the variance_decomposition() function in the performance package^65^ to calculate repeatability estimates for bayesian models from posterior predictive distributions. Spearman’s rank correlations were calculated with the cor.test() function in the stats package included in R^45^ (table reproduced from Creamer & Horback^37^).

Measurement	Repeatability (*R*)		CI Repeatability	
Handle duration (s)	0.60		[0.30, 0.79]	
Chute duration (s)	0.69		[0.42, 0.84]	
Squeeze duration (s)	0.76		[0.47, 0.90]	
Exit duration (s)	0.72		[0.37, 0.90]	
Latency to supplement	Social-Feed Trade-off (12 m)		Novel bucket (6 m)	
	rho	p	rho	p
	0.353	0.015	0.559	< 0.001

#### Grazing-related metrics models

We used the glmmTMB package in R^45,66^ to run mixed models for analysis of these data. We ran eight separate models for the six weekly-averaged rangeland use metrics and the two full-season metrics (average elevation, average daily distance traveled, average slope, average distance to water, average distance to loafing sites, average distance to supplement sites, adjusted kernel density 50% core range [one measure per year], social network strength [one measure per year]). The fixed effects in statistical models accounted for year, week (only for weekly averaged measures, not for the kernel density estimate nor social network strength), age of cows, and temperature (again only for average weekly measures) and included the four behavior measures from the first repetition per year of the management assay, latency to familiar supplement for each year, and latency to novel bucket for each year as fixed effects. Year as a fixed effect in these models not only accounted for possible climatic conditions that differed between years, but also the addition of the new water trough at higher elevation in year two. We used a second-order polynomial term for week to account for temporal autocorrelation where weeks closer together in time are more similar to each other^47,67^. Models contained a random effect of cow ID to account for repeated measures across weeks and years of individual cows.

We centered and scaled predictor variables to standard deviations (i.e. standardized) to aid in the interpretation of relative effect of the predictor variables on the rangeland use metrics^68^ except we did not scale year or week because they were temporal components in our models. We checked collinearity on a linear model of the same predictor variables with the vif function in the car package^69^, all VIFs (variance inflation factor) were around 1, indicating no issues with collinearity between predictors.

We modified additional model parameters that improved model fit and the residuals *versus* predicted plots, like setting the dispersion parameter (dispformula) to year and transforming skewed variables to the log scale. We checked models via visualizing QQ plots from simulated residuals in the DHARMa package^70^ and residual *versus* predicted plots of the models, which did not show any obvious patterns in the residuals. The slope, loaf, and distance model had some outliers flagged in the DHARMa QQ plots, however we did not take these outliers out as the GPS data had already gone through cleaning and processing. The DHARMa package also flagged deviation as significant for the slope model (*p* = 0.038), however when we visually inspected the QQ simulated residual plot, the deviation seemed minor and could probably be attributed to the large data set. Significance level was set at α = 0.05, statistical trends are reported if (0.05 < *p* < 0.10).

## Results

### Relationships between assay-related metrics and grazing-related metrics

The time it took humans to move cows into the chute, as well as the time the cow took to exit the hydraulic squeeze, did not predict any rangeland use metrics (Table 3; Supplemental Table 1). In contrast, the behavior of cows once inside the chute did relate to a few rangeland metrics; more passive cows in the chute ranged higher and were more expansive in their rangeland use. For both years, cows that took longer to traverse the cement chute traveled higher in elevation on range (*p* = 0.017), were further from water (*p* = 0.043), and closer to supplement sites (*p* = 0.029), and also tended to use steeper slopes (*p* = 0.060) and travel wider areas (*p* = 0.067; Table 3; Supplemental Table 1). The duration in the cement chute did not significantly predict distance traveled, distance to loafing sites, nor social network strength. Cows that took longer to traverse the hydraulic squeeze also tended to be closer to loafing sites (*p* = 0.076) and have lower social network strength (*p* = 0.095) for both years (Table 3; Supplemental Table 1). Time to traverse the hydraulic squeeze did not significantly predict any rangeland use metrics. Cows that had higher latencies to supplement in the social-feed trade-off assay (SFTA) traveled shorter daily distances on rangeland (*p* = 0.035) for both years (Table 3; Supplemental Table 1). Latency to supplement in the social-feed trade-off assay did not predict other rangeland use metrics besides distance traveled. The latency to supplement in the novel approach assay did not significantly predict any rangeland use metrics (Table 3; Supplemental Table 1).

### Effect of temporal, environmental, and animal variables on grazing-related metrics

**Fig. 4:**
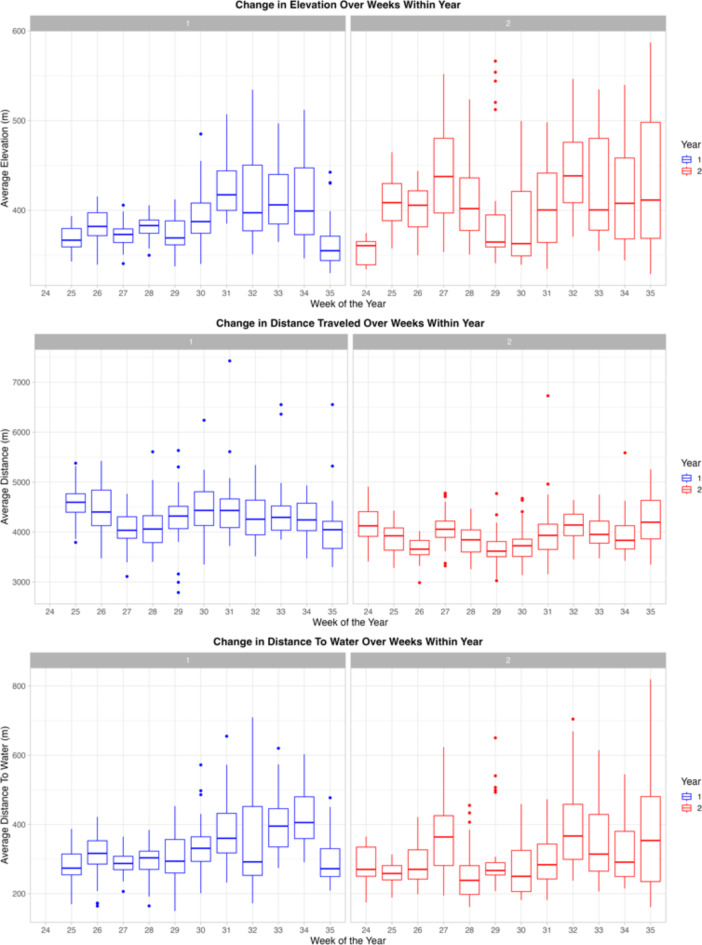
The change over time (across week and year) in three of the grazing-related metrics collected over two consecutive summers (*n* = 47): TOP: Elevation, MIDDLE: Distance traveled, BOTTOM: Distance to water.

In comparison to year one, cows were recorded to be at higher elevations (*p* < 0.001), traveled shorter distances (*p* < 0.001), were closer to water (*p* = 0.002), supplement (*p* < 0.001), and loafing sites (*p* = 0.048) in year two (Fig. 4). In addition, cows also had lower social network degree strengths in year two than in year one (statistical trend, *p* = 0.051). This indicates that there was weak evidence that cows had lower cohesion (less proximity to conspecifics) while on rangeland in year two. For both years, cows expanded their location across the pasture over the weeks (Fig. 4); being found at higher elevations (*p* < 0.001), on steeper slopes (*p* < 0.001), further from water (*p* < 0.001) and closer to supplement sites (*p* < 0.001), and they traveled further distances (*p* < 0.001). Higher average temperature meant cows used more gradual slopes (*p* < 0.001), were closer to water (*p* < 0.001), and traveled shorter distances (*p* < 0.001), and there was weaker evidence they also used lower elevations (*p* = 0.061). Average temperature did not influence distance to supplement or loafing sites. In both years, older cows used areas closer to loafing sites (*p* = 0.022), and there was a trend for older cows to also use areas closer to supplement (*p* = 0.078). Age of cattle did not affect other rangeland use metrics.

**Table 3:** A summary of the statistically significant relationships found among the assay-related and grazing-related metrics.

Grazing-related metrics	Year	Week	Temp	Age	Handle	Chute	Squeeze	Latency to Familiar Supplement	Latency to Novel Supplement
Elevation (log)	+ 0.045***	+ 0.009***	-0.006			+ 0.014*			
Slope (log)		+ 0.011***	-0.018***			+ 0.013			
Distance Traveled (log)	-0.110***	+ 0.004***	-0.028***					-0.011*	
Distance to supplement	-150.948***	-14.178***		-19.110		-20.059*			
Distance to water (log)	-0.070**	+ 0.031***	-0.066***			+ 0.030*			
Distance to loaf	-5.800*			-3.958*			-2.691		
AKDE 50%	-4.402					+ 2.349			
SN Degree Strength	-0.106						-0.046		

A plus sign indicates a positive change in the response (positive estimate), and negative sign indicates a negative change in the response (negative estimate) per unit change of the independent variables listed. Log estimates and *p* values (where *0.01 < *p* < 0.05, **p < 0.01, ***p < 0.001, and estimates without an asterisk indicate a trend of 0.05 < *p* < 0.10) are provided. Boxes without estimates demonstrate non-significant relationships between independent variables and grazing-related metrics. Further information about models and all estimates can be found in the supplemental Table 1.

## Discussion

This study was the first to relate consistent individual differences (CIDs) in beef cattle behavior measured in a management assay, and feeding preferences in trade-off assays, to grazing patterns on extensive rangeland. The results of this research provide evidence that cows which display reduced activity during isolation in a management assay traveled to higher elevations and further from water during two summer grazing seasons in the Sierra Nevada foothills. Feeding behavior in a social-feed trade-off assay predicted distance traveled while cattle were on rangeland (a group setting with minimal human interference). There was no evidence that handling durations nor exit duration related to grazing behavior while cows were on rangeland, but weak evidence that behavior while cows were in the hydraulic squeeze predicted closer proximity to loafing sites and less social proximity while on rangeland.

### Cows altered grazing patterns over time

In year two, cows used areas at higher elevation, traveled shorter distances and were closer to water, supplement and loafing sites (see ‘Effect of Temporal, Environmental, and Animal Variables on Grazing-Related Metrics’), which may be explained by the addition of a new water source at higher elevations (Fig. 1). Walburger et al.^71^ concludes that water is one of the most influential factors shaping grazing distribution in herds of cattle, so it is not surprising that, overall, cattle altered grazing patterns in year two to accommodate a new water source. These results reflect group-level differences in behavior, however it should be noted that we have evidence that individual cattle grazing patterns remained *relatively* (as in relative among cattle) consistent across years^38^.

Cows expanded grazing patterns as weeks passed within the grazing season. Cows were using higher elevation, were on more rugged terrain, and were farther from water sites as weeks passed within the grazing season likely because preferred grazing areas were depleted of quality vegetation^72^, and cows had to travel farther away to graze where vegetation was readily available^73–75^. Providing diversified management tools and strategic placement of resources, and if feasible doing this dynamically throughout a season, could contribute to improved grazing distribution within and across seasons.

Hotter temperatures meant cows prioritized water and conserved their energy exertion by not traveling far from water sites, nor traveling as far, as high, or on rugged terrain which makes intuitive sense and has also been found in several studies [^76,77^, reviewed by 75]. Older cows used areas on rangeland closer to supplement and closer to loafing sites. Several other studies have also found older cows to consume more supplement^78,79^, and remain closer to supplement during grazing^80^ than younger cows.

### Less active cows in the chute have more optimal grazing distribution

Cows that took longer to traverse the initial part of the working chute (a cement alleyway), showing a more passive response to the assay and to isolation, also exhibited more optimal grazing behavior by traveling higher in elevation, further from water, closer to supplement (most placed at higher areas on range, Fig. 1) and they tended to have larger core home ranges and use more rugged terrain. Although we did not collect measurements to determine coping styles per se, the duration it took cows to traverse areas of the chute (a stressful and isolated context) may have been a measure of stress coping in cows. Proactive and reactive coping styles are ways to classify livestock animals, most commonly used with pigs^11^, wherein proactive individuals show a more active, routine response to stress and less HPA axis reactivity and reactive individuals show a more passive, cautious response to stress and have higher HPA axis reactivity^81–83^. While we did not measure physiological response to handling and isolation to support our claims that the animals were experiencing stress, cows that took longer in the cement alley were also more stationary^37^, thus may be exhibiting a reactive coping style^81^. Reactive individuals have been found to exhibit more behavioral flexibility in changing environments^84–87^ than their proactive counterparts. This may explain why cows which are assumed to display reactive coping in the chute exhibit more optimal grazing patterns on a complex, rangeland environment where social groups and access to quality vegetation varies throughout the season. This would also explain why they demonstrate flexibility in traveling further from water, higher on range, and why they used upland supplement that they had to explore the range to discover.

Culling female breeding cattle from the herd is driven by reproductive and production traits; some of the primary reasons to cull cattle are for fertility issues, aborting calves, difficulty calving (all which can occur with older age), and low weaning weights of calves^88^. In our study, we did not explore the relationship between reproduction traits, behavior, and grazing, but we did find some evidence that age and behavior relate to grazing patterns. Only a few studies in cattle have looked at relationships between reproductive traits and behavior (e.g^89^). or reproduction and grazing. Given that reproductive health and production traits are the driving reasons to cull cows from the herd, and thus shaping future grazers on rangeland, more research needs to be done on the relationship between behavior, coping styles, reproductive traits, and grazing. In a few cases, ranchers cull based on behavior traits, but that is often if cattle are extremely aggressive and difficult to work with (for safety and animal welfare concern;^90^). Some cows have larger, more sensitive, flight zones and react more actively to handling interventions, while others may show a more passive response^90^. If ranchers cull cattle that are ‘stubborn’ or less active upon moving or sorting, they may unintendedly be shaping a less optimal distribution of their animals on rangeland by culling those that graze higher elevations, are finding upland supplement, and are not clumping near water. We found that behavior of cattle in the chute does predict some grazing patterns, which can be useful in shaping a herd’s grazing utilization by selecting cows with specific observable behaviors to be more optimal grazers. Shaping the environment, for example adding targeted supplements^91^, may improve grazing distribution of the herd to some degree. However, our results suggest that optimized grazing may be enhanced by cows with specific behavior types, such as those more passive in the chute and more flexible in their grazing patterns. If ranchers apply both grazing improvements by selecting cows more passive in the chute and adding targeted supplements, they may see much wider grazing distribution than they would by just adding supplement.

### Potential applications of assay-related behaviors to alter grazing behavior

Latency to supplement in the social-feed trade-off task represented a consistent feeding behavior such that cows that were less feed-centric during the task, with longer latencies to the supplement, also traveled less on rangeland to forage (similar to findings by Wesley et al.^30^, ; Goodman et al.^31^, with supplement consumption rate). Cows with longer latencies to supplement were presumably less food-motivated or less willing to travel away from their social group to consume food or supplement^4,92,93^. Ranchers often want to retain cattle in the herd that travel farther away on rangeland and are more willing to separate from herd mates to do so because they are better maintaining vegetation and soil health, and not clumping in the same locations^94–96^. Observing a cow’s willingness to consume supplement at least 12 m away from conspecifics could enable ranchers to predict how cattle will travel to forage on rangeland.

Neither handle duration, exit duration, nor the novel approach assay predicted behaviors on rangeland. It was perhaps not surprising that handle duration did not relate to grazing-related metrics because this relied on subjectivity of human handling and uncontrollable reactions from groupmates that were in the corral. It is, however, surprising the exit duration did not relate to any grazing-related metrics as this is a widely accepted measurement of temperament in cattle^12,16^ and has been found to relate to a multitude of behaviors in cattle (like social behavior^15^, and feeding behavior^21^).

We assumed this novel approach assessment would relate to grazing-related metrics because it involved a practical social *versus* novelty trade-off that cattle are likely to face especially while on new pastures. However, novel object tests in cattle have been used in a variety of experiments and have not related to other behaviors in some studies^37,97,98^. We used novel color and visual patterns on the bucket that were species-specific in terms of cattle vision^99^, but not in terms of what they might confront on rangeland. This assessment could be modified to include novel, but natural, stimuli like a novel vegetation species (carrots have been used in dairy cattle;^100^) or a bucket surrounded with novel logs or rocks.

Studies that measure consistent individual differences across manipulated experiments and in field settings are rare and crucial^101^, and overcome methodological hurdles of validating representative behavior traits in animals. It has been expressed in studies and surveys that allowing cattle to habituate to handling and move through chutes unrestrained^102^ will promote more efficiency and better animal welfare in future processing procedures in cattle (breeding, health checks, vaccinations, etc.;^103,104^). If cattle managers can observe behavior of cattle during this situation of minimal interference in the narrow chute while habituating animals, they may be able to identify passive cows that are likely to exhibit more optimal foraging patterns. Our behavior measures were from the first day of data of each year because it is likely only one exposure to assessments would be used on working ranches to assess temperament^34,105^. Identifying desirable grazing characteristics of cattle and understanding cattle social behaviors could allow ranchers to shape herds constituting certain individuals or strategically plan certain management tools like targeted supplement^91,94,106^.

### Limitations

Our results presented here are based on durations rather than velocity or speed, as these are proportional and do not affect the differences between individuals, however we recognize this might be difficult to generalize across facilities. We have included the distances of the cement chute, hydraulic squeeze chute, and exit area (see ethogram Table 1) so that researchers may make their own calculations of speed to compare results. We also recognize that some of our reported results are statistical trends rather than below the threshold of statistical significance, but we are reporting all relevant results for this exploratory study, rather than strictly ‘statistically relevant’ for transparency and to encourage future exploration of these relationships^107,108^.

### Conclusion and implications

Cows that were less active in a narrow cement chute were found to have more optimal grazing distribution for rangeland conservation by using higher elevation, grazing further from water, and closer to upland supplement. Cows with higher latencies to a familiar supplement in an experimental social-feed trade-off task did not travel as far on rangeland to forage. Optimal grazing on extensive rangeland provides a suite of direct benefits to humans, animals, and the environment^29^. There is discernable potential for unmanaged or unchecked cattle grazing to negatively interfere with rangeland benefits and result in a host of issues ranging from diminished water quality to degradation of important habitats for a variety of ecosystems^73,94^. There is evidence that duration to traverse the chute and latency to a familiar supplement both are cross-contextual measures relating to behaviors on rangeland. Observable consistent individual differences in cattle behavior during handling and management procedures that predict grazing patterns has potential to inform ranchers on shaping cattle distribution to achieve rangeland conservation goals and avoid the negative impacts of uneven grazing.

## Electronic supplementary material

Below is the link to the electronic supplementary material.

Supplementary Material 1

Supplementary Material 2

Supplementary Material 3

Supplementary Material 4

Supplementary Material 5

## Data Availability

Data and R code are available in supplemental materials.
