# Supplementary material for: Cows that are less active in the chute have more optimal grazing distribution
Source: Sci Rep. 2025 Jan 2;15:58. doi: 10.1038/s41598-024-84090-z (PMC11696303; doi:10.1038/s41598-024-84090-z)
Supplement: Supplementary file 2 — Supplementary Material 2 [file 41598_2024_84090_MOESM2_ESM.docx]

Dryad metadata: <https://datadryad.org/docs/README.md>

- Summary of experimental efforts underlying this dataset
- Description of file structure and contents
- Definitions of all variables, abbreviations, missing data codes, and units
- Links to other publicly accessible locations of the data
- Other sources, if any, that the data was derived from
- Any other details that may influence reuse or replication efforts

This dataset contains GPS data of rangeland cattle over two summer grazing seasons (2021 and 2022) aggregated by week of daily averages of rangeland use metrics. Rangeland use metrics include elevation used, slope used, distance traveled, distance to supplement sites, distance to loafing sites, and distance to water sites. It also includes values for adjusted kernel density 50% home range and social network degree strength, both of which are a single value for the grazing season (within year). Cows that were tracked also participated in behavior assays (see Creamer & Horback, 2024, in review) and behaviors from these assays are included in the dataset as well. Age of the cows while grazing (in years) and temperature data (in F) are included as well. All predictor variables in models (age, temperature, behavior variables) are mean centered and scaled to standard deviation.

GPS data has been cleaned and processed for outliers and extreme values. NAs in GPS data indicate that the collar stopped fixing on satellites, died, or fell off by that time. NAs in the behavior datasets indicate that that particular animal did not complete behavior testing across repetitions of the management assay (see Creamer & Horback, 2024, in review)

Subjects: Cow ID

Year: Year 1 (2021) and 2 (2022) when data was taken

mean_elev: average daily elevation aggregated by week (mean)

mean_slope: average daily slope aggregated by week (mean)

min_temp:average daily minimum temperature aggregated by week (mean)

max_temp: average daily maximum temperature aggregated by week (mean)

avgtemp: average daily temperature aggregated by week (mean) (raw)

avgtemp.s: average daily temperature aggregated by week (mean) (scaled)

Age: Age of cows (in years)

Age.s: Age of cows centered by mean and scaled by standard deviation

mean_water: average daily distance to water aggregated by week (mean)

mean_loaf: average daily distance to loaf sites aggregated by week (mean)

mean_supp: average daily distance to supplement aggregated by week (mean)

dist.avg: daily distance traveled aggregated by week (mean)

strength: social network degree strength across entire grazing season

homerange.50: adjusted kerned density estimate of 50% home range across entire grazing season

week: week of the year

uniqueweek: week of the year and year connected with underscore (_)

handtotalttb_d1: duration to handle into chute day 1 in seconds (raw)

chcem_alley_d1: duration to traverse cement chute day 1 in seconds (raw)

se_squeeze_d1: duration to traverse hydraulic squeeze day 1 in seconds (raw)

se_exit_d1: duration to exit hydraulic squeeze day 1 in seconds (raw)

sftlat_supp: latency to approach supplement on 12m bucket distance day in whole seconds (raw)

nsftlat_supp: latency to approach novel supplement bucket in whole seconds (raw)

sftlat_supp.s: latency to approach supplement on 12m bucket distance day in whole seconds (scaled)

nsftlat_supp.s: latency to approach novel supplement bucket in whole seconds (scaled)

chcem_alley_d1.s: duration to traverse cement chute day 1 in seconds (scaled)

handtotalttb_d1.s: duration to handle into chute day 1 in seconds (scaled)

se_squeeze_d1.s: duration to traverse hydraulic squeeze day 1 in seconds (scaled)

se_exit_d1.s: duration to exit hydraulic squeeze day 1 in seconds (scaled)

Code used on finalized data showing models that were run, model checks of residuals, visualizations of data, and tables created for the manuscript are included.
