# Supplementary material for: Cows that are less active in the chute have more optimal grazing distribution
Source: Sci Rep. 2025 Jan 2;15:58. doi: 10.1038/s41598-024-84090-z (PMC11696303; doi:10.1038/s41598-024-84090-z)
Supplement: Supplementary file 3 — Supplementary Material 3 [file 41598_2024_84090_MOESM3_ESM.docx]

Supplemental Table 1. Results of models for each rangeland metric: elevation, slope, distance traveled, distance to water, distance to supplement sites, distance to loaf sites, adjusted kernel density estimate of 50% home range, and social network degree strength. All predictor variables except year and week were centered and standardized, thus effects on response variables represent relative effects across predictors. Models that were run with log transformed versus Gaussian response variables are specified as such in the table. Estimates of log-transformed response variables in the table are on the log scale and have not been back-transformed. Results for model parameters which *p*<0.05 are bolded, tendencies (0.05<*p*<0.10) are indicated with an asterisk *.

| **Elevation^y^** | | | | |
| --- | --- | --- | --- | --- |
|  | **Elevation (log, meters)** | | | |
| *Predictors* | *Estimates* | *std. Error* | *Statistic* | *p* |
| Intercept | 5.691 | 0.031 | 184.740 | **<0.001** |
| **Year (2)** | 0.045 | 0.009 | 5.234 | **<0.001** |
| **Week** | 0.009 | 0.001 | 9.037 | **<0.001** |
| Avg temp (F)* | -0.006 | 0.003 | -1.874 | 0.061* |
| Age | 0.011 | 0.008 | 1.417 | 0.157 |
| Handle duration | -0.002 | 0.004 | -0.462 | 0.644 |
| **Chute duration** | 0.014 | 0.006 | 2.392 | **0.017** |
| Squeeze duration | -0.003 | 0.005 | -0.675 | 0.500 |
| Exit duration | -0.001 | 0.005 | -0.233 | 0.816 |
| Latency to familiar supplement | -0.006 | 0.005 | -1.144 | 0.252 |
| Latency to novel supplement | -0.005 | 0.006 | -0.771 | 0.441 |
| **Slope^y^** | | | | |
|  | **Slope (log, degrees)** | | | |
| *Predictors* | *Estimates* | *std. Error* | *Statistic* | *p* |
| Intercept | 1.878 | 0.041 | 46.245 | **<0.001** |
| Year (2) | -0.013 | 0.011 | -1.215 | 0.225 |
| **Week** | 0.011 | 0.001 | 8.522 | **<0.001** |
| **Avg temp (F)** | -0.018 | 0.004 | -4.166 | **<0.001** |
| Age | -0.002 | 0.007 | -0.339 | 0.735 |
| Handle duration | -0.001 | 0.005 | -0.262 | 0.793 |
| Chute duration* | 0.013 | 0.007 | 1.878 | 0.060* |
| Squeeze duration | -0.004 | 0.005 | -0.762 | 0.446 |
| Exit duration | -0.009 | 0.006 | -1.607 | 0.108 |
| Latency to familiar supplement | 0.004 | 0.006 | 0.594 | 0.552 |
| Latency to novel supplement | 0.002 | 0.006 | 0.272 | 0.786 |
| **Distance Traveled** | | | | |
|  | **Distance Traveled (log, meters)** | | | |
| *Predictors* | *Estimates* | *std. Error* | *Statistic* | *p* |
| Intercept | 8.242 | 0.033 | 253.136 | **<0.001** |
| **Year (2)** | -0.110 | 0.009 | -12.080 | **<0.001** |
| **Week** | 0.004 | 0.001 | 4.201 | **<0.001** |
| **Avg temp (F)** | -0.028 | 0.004 | -8.040 | **<0.001** |
| Age | 0.005 | 0.007 | 0.645 | 0.519 |
| Handle duration | 0.000 | 0.004 | 0.004 | 0.997 |
| Chute duration | -0.003 | 0.006 | -0.439 | 0.660 |
| Squeeze duration | -0.000 | 0.005 | -0.084 | 0.933 |
| Exit duration | 0.003 | 0.005 | 0.709 | 0.478 |
| **Latency to familiar supplement** | -0.011 | 0.005 | -2.105 | **0.035** |
| Latency to novel supplement | 0.004 | 0.006 | 0.707 | 0.479 |
| **Distance to Supplement^y^** | | | | |
|  | **Distance to Supplement (Gaussian, meters)** | | | |
| *Predictors* | *Estimates* | *std. Error* | *Statistic* | *p* |
| Intercept | 1070.951 | 54.981 | 19.478 | **<0.001** |
| **Year (2)** | -150.948 | 14.728 | -10.249 | **<0.001** |
| **Week** | -14.178 | 1.806 | -7.849 | **<0.001** |
| Avg temp (F) | 2.006 | 6.074 | 0.330 | 0.741 |
| Age* | -19.110 | 10.853 | -1.761 | 0.078* |
| Handle duration | 2.982 | 7.049 | 0.423 | 0.672 |
| **Chute duration** | -20.059 | 9.202 | -2.180 | **0.029** |
| Squeeze duration | 8.888 | 7.985 | 1.113 | 0.266 |
| Exit duration | 0.258 | 7.635 | 0.034 | 0.973 |
| Latency to familiar supplement | 4.619 | 8.229 | 0.561 | 0.575 |
| Latency to novel supplement | 7.275 | 9.369 | 0.776 | 0.437 |
| **Distance to Water** | | | | |
|  | **Distance to Water (log, meters)** | | | |
| *Predictors* | *Estimates* | *std. Error* | *Statistic* | *p* |
| Intercept | 4.843 | 0.080 | 60.465 | **<0.001** |
| **Year (2)** | -0.070 | 0.022 | -3.120 | **0.002** |
| **Week** | 0.031 | 0.003 | 11.981 | **<0.001** |
| **Avg temp (F)** | -0.066 | 0.009 | -7.664 | **<0.001** |
| Age | 0.029 | 0.020 | 1.439 | 0.150 |
| Handle duration | -0.002 | 0.011 | -0.171 | 0.864 |
| **Chute duration** | 0.030 | 0.015 | 2.024 | **0.043** |
| Squeeze duration | 0.002 | 0.012 | 0.139 | 0.889 |
| Exit duration | -0.017 | 0.011 | -1.507 | 0.132 |
| Latency to familiar supplement | -0.007 | 0.013 | -0.547 | 0.585 |
| Latency to novel supplement | -0.007 | 0.016 | -0.433 | 0.665 |
| **Distance to Loaf** | | | | |
|  | **Distance to Loaf (Gaussian, meters)** | | | |
| *Predictors* | *Estimates* | *std. Error* | *Statistic* | *p* |
| Intercept | 130.268 | 11.452 | 11.376 | **<0.001** |
| **Year (2)** | -5.800 | 2.931 | -1.979 | **0.048** |
| Week | 0.005 | 0.379 | 0.012 | 0.990 |
| Avg temp (F) | 0.060 | 1.259 | 0.047 | 0.962 |
| **Age** | -3.958 | 1.727 | -2.291 | **0.022** |
| Handle duration | -0.798 | 1.385 | -0.576 | 0.565 |
| Chute duration | 0.016 | 1.623 | 0.010 | 0.992 |
| Squeeze duration* | -2.691 | 1.514 | -1.777 | 0.076* |
| Exit duration | -1.002 | 1.418 | -0.707 | 0.480 |
| Latency to familiar supplement | 0.101 | 1.554 | 0.065 | 0.948 |
| Latency to novel supplement | -1.541 | 1.600 | -0.963 | 0.336 |
| **AKDE50** | | | | |
|  | **Area of 50% home range (Gaussian, hectares)** | | | |
| *Predictors* | *Estimates* | *std. Error* | *Statistic* | *p* |
| Intercept | 73.213 | 4.368 | 16.760 | **<0.001** |
| Year (2)* | -4.402 | 2.651 | -1.660 | 0.097* |
| Age | -0.278 | 1.235 | -0.225 | 0.822 |
| Handle duration | -0.555 | 1.106 | -0.502 | 0.616 |
| Chute duration* | 2.349 | 1.281 | 1.833 | 0.067* |
| Squeeze duration | -1.924 | 1.227 | -1.568 | 0.117 |
| Exit duration | 0.870 | 1.191 | 0.730 | 0.465 |
| Latency to familiar supplement | -0.137 | 1.257 | -0.109 | 0.913 |
| Latency to novel supplement | 1.361 | 1.228 | 1.108 | 0.268 |
| **Social Network Strength** | | | | |
|  | **SN Strength (Gaussian)** | | | |
| *Predictors* | *Estimates* | *std. Error* | *Statistic* | *p* |
| Intercept | 1.591 | 0.091 | 17.480 | **<0.001** |
| Year (2)* | -0.106 | 0.054 | -1.948 | 0.051* |
| Age | -0.025 | 0.029 | -0.886 | 0.376 |
| Handle duration | -0.013 | 0.024 | -0.552 | 0.581 |
| Chute duration | 0.006 | 0.029 | 0.200 | 0.842 |
| Squeeze duration* | -0.046 | 0.027 | -1.671 | 0.095* |
| Exit duration | 0.006 | 0.026 | 0.212 | 0.832 |
| Latency to familiar supplement | -0.012 | 0.027 | -0.450 | 0.653 |
| Latency to novel supplement | 0.040 | 0.027 | 1.476 | 0.140 |

**^y^** Models were run with dispformula = ~ year to account for heteroscedasticity in residuals.
