# Supplementary material for: Cows that are less active in the chute have more optimal grazing distribution
Source: Sci Rep. 2025 Jan 2;15:58. doi: 10.1038/s41598-024-84090-z (PMC11696303; doi:10.1038/s41598-024-84090-z)
Supplement: Supplementary file 5 — Supplementary Material 5 [file 41598_2024_84090_MOESM5_ESM.docx]

Gps x Behavior Analysis

2024-01-07

#### Libraries

library(ggplot2)
library(dplyr)
library(ggeffects)
library(car)
library(glmmTMB)
library(DHARMa)
library(sjPlot)
library(lme4)

# Data

## Data across weeks

dat.week <- read.csv("~/Library/CloudStorage/GoogleDrive-mlcreame@ncsu.edu/My Drive/SciReportsPaperSubmission/dat.week.csv") #includes raw data and standardized data (mean centered and scaled by standard deviation)

## Data for AKDE 50 home range and social network degree strength (one value per season)

akde.sn.yr <- dat.week %>%
 filter(uniqueweek == '1_25'|uniqueweek == '2_24')

### Factor variables

dat.week$year <- as.factor(dat.week$year)
dat.week$Subjects <- as.factor(dat.week$Subjects)

# Models

### Slope

slope <- glmmTMB(log(mean_slope) ~ year + week + week^2 + avgtemp.s + Age.s + chcem_alley_d1.s + handtotalttb_d1.s + se_exit_d1.s + se_squeeze_d1.s + sftlat_supp.s + nsftlat_supp.s + (1|Subjects), data = dat.week, dispformula = ~year) #improves heteroskedasticity
summary(slope)

## Family: gaussian ( identity )
## Formula: log(mean_slope) ~ year + week + week^2 + avgtemp.s + Age.s +
## chcem_alley_d1.s + handtotalttb_d1.s + se_exit_d1.s + se_squeeze_d1.s +
## sftlat_supp.s + nsftlat_supp.s + (1 | Subjects)
## Dispersion: ~year
## Data: dat.week
##
## AIC BIC logLik deviance df.resid
## -1111.9 -1044.9 570.0 -1139.9 870
##
## Random effects:
##
## Conditional model:
## Groups Name Variance Std.Dev.
## Subjects (Intercept) 0.001484 0.03853
## Residual NA NA
## Number of obs: 884, groups: Subjects, 49
##
## Conditional model:
## Estimate Std. Error z value Pr(>|z|)
## (Intercept) 1.878054 0.040611 46.25 < 2e-16 ***
## year2 -0.012863 0.010590 -1.21 0.2245
## week 0.011428 0.001341 8.52 < 2e-16 ***
## avgtemp.s -0.018478 0.004435 -4.17 3.1e-05 ***
## Age.s -0.002492 0.007358 -0.34 0.7349
## chcem_alley_d1.s 0.012812 0.006821 1.88 0.0603 .
## handtotalttb_d1.s -0.001309 0.004989 -0.26 0.7930
## se_exit_d1.s -0.008993 0.005596 -1.61 0.1080
## se_squeeze_d1.s -0.004170 0.005473 -0.76 0.4461
## sftlat_supp.s 0.003553 0.005981 0.59 0.5525
## nsftlat_supp.s 0.001763 0.006489 0.27 0.7859
## ---
## Signif. codes: 0 '***' 0.001 '**' 0.01 '*' 0.05 '.' 0.1 ' ' 1
##
## Dispersion model:
## Estimate Std. Error z value Pr(>|z|)
## (Intercept) -2.28368 0.04075 -56.04 < 2e-16 ***
## year2 0.31369 0.05220 6.01 1.87e-09 ***
## ---
## Signif. codes: 0 '***' 0.001 '**' 0.01 '*' 0.05 '.' 0.1 ' ' 1

res.slope = simulateResiduals(slope, n = 2000)
plotQQunif(res.slope) #visual inspection looks ok, deviation attributed to large sample size


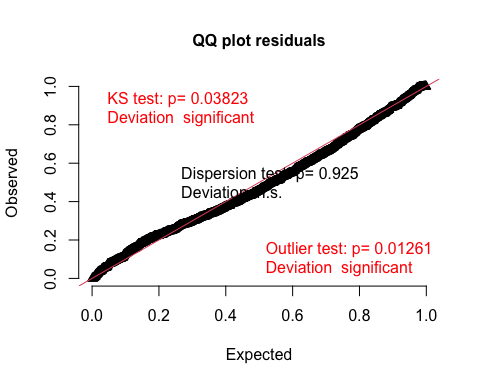


resid.slope <- resid(slope)
pred.slope <- predict(slope)
hist(resid.slope)


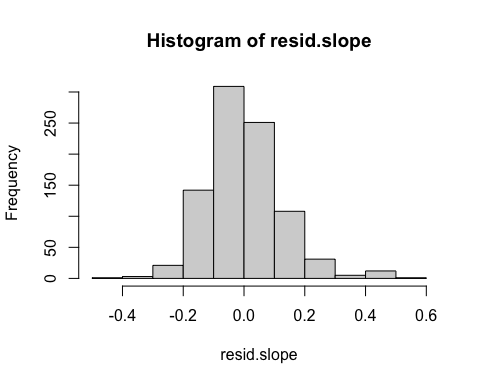


plot(pred.slope, resid.slope)


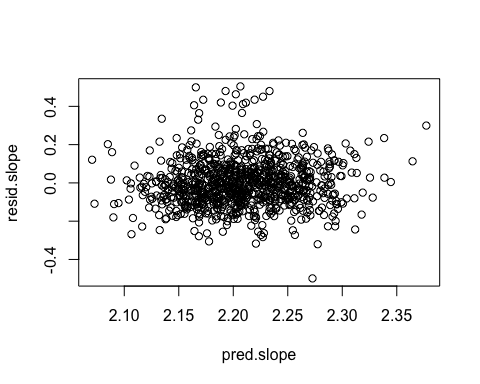


#tab_model(slope, show.se = TRUE, show.ci = FALSE, show.stat = TRUE, digits = 3, digits.p = 3)

### Distance traveled

dist <- glmmTMB(log(dist.avg) ~ year + week + week^2 + avgtemp.s + Age.s + chcem_alley_d1.s + handtotalttb_d1.s + se_exit_d1.s + se_squeeze_d1.s + sftlat_supp.s + nsftlat_supp.s + (1|Subjects), data = dat.week)
summary(dist)

## Family: gaussian ( identity )
## Formula:
## log(dist.avg) ~ year + week + week^2 + avgtemp.s + Age.s + chcem_alley_d1.s +
## handtotalttb_d1.s + se_exit_d1.s + se_squeeze_d1.s + sftlat_supp.s +
## nsftlat_supp.s + (1 | Subjects)
## Data: dat.week
##
## AIC BIC logLik deviance df.resid
## -1538.2 -1476.0 782.1 -1564.2 870
##
## Random effects:
##
## Conditional model:
## Groups Name Variance Std.Dev.
## Subjects (Intercept) 0.001835 0.04284
## Residual 0.009163 0.09573
## Number of obs: 883, groups: Subjects, 49
##
## Dispersion estimate for gaussian family (sigma^2): 0.00916
##
## Conditional model:
## Estimate Std. Error z value Pr(>|z|)
## (Intercept) 8.242e+00 3.256e-02 253.14 < 2e-16 ***
## year2 -1.100e-01 9.104e-03 -12.08 < 2e-16 ***
## week 4.455e-03 1.060e-03 4.20 2.65e-05 ***
## avgtemp.s -2.826e-02 3.514e-03 -8.04 8.96e-16 ***
## Age.s 4.735e-03 7.338e-03 0.65 0.5187
## chcem_alley_d1.s -2.506e-03 5.702e-03 -0.44 0.6603
## handtotalttb_d1.s 1.582e-05 4.425e-03 0.00 0.9971
## se_exit_d1.s 3.206e-03 4.520e-03 0.71 0.4781
## se_squeeze_d1.s -4.116e-04 4.907e-03 -0.08 0.9332
## sftlat_supp.s -1.084e-02 5.150e-03 -2.11 0.0353 *
## nsftlat_supp.s 4.261e-03 6.026e-03 0.71 0.4794
## ---
## Signif. codes: 0 '***' 0.001 '**' 0.01 '*' 0.05 '.' 0.1 ' ' 1

res.dist = simulateResiduals(dist, n = 2000) #outliers but ok given processing of data
plotQQunif(res.dist)


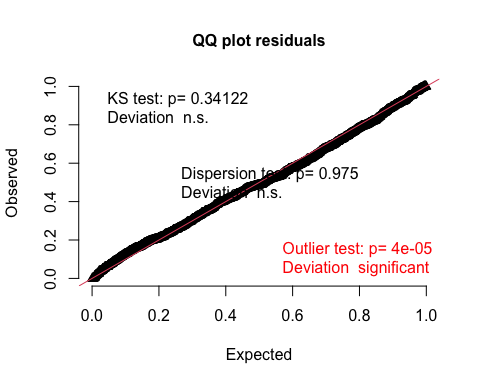


resid.dist <- resid(dist)
pred.dist <- predict(dist)
hist(resid.dist)


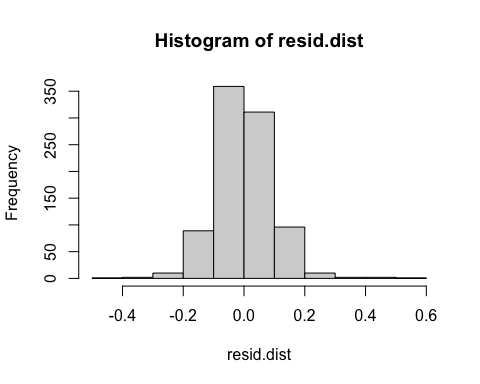


plot(pred.dist, resid.dist)


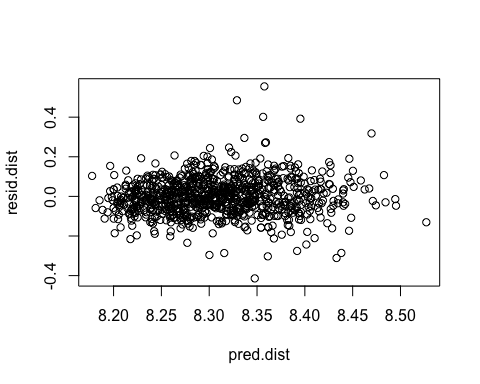


#tab_model(dist, show.se = TRUE, show.ci = FALSE, show.stat = TRUE, digits = 3, digits.p = 3)

### Elevation

elev <- glmmTMB(log(mean_elev) ~ year + week + week^2 + avgtemp.s + Age.s + chcem_alley_d1.s + handtotalttb_d1.s + se_exit_d1.s + se_squeeze_d1.s + sftlat_supp.s + nsftlat_supp.s + (1|Subjects), data = dat.week, dispformula = ~year) #dispformula year improves heteroskedasticity in residuals

summary(elev)

## Family: gaussian ( identity )
## Formula:
## log(mean_elev) ~ year + week + week^2 + avgtemp.s + Age.s + chcem_alley_d1.s +
## handtotalttb_d1.s + se_exit_d1.s + se_squeeze_d1.s + sftlat_supp.s +
## nsftlat_supp.s + (1 | Subjects)
## Dispersion: ~year
## Data: dat.week
##
## AIC BIC logLik deviance df.resid
## -1564.2 -1497.2 796.1 -1592.2 870
##
## Random effects:
##
## Conditional model:
## Groups Name Variance Std.Dev.
## Subjects (Intercept) 0.002041 0.04518
## Residual NA NA
## Number of obs: 884, groups: Subjects, 49
##
## Conditional model:
## Estimate Std. Error z value Pr(>|z|)
## (Intercept) 5.691126 0.030806 184.74 < 2e-16 ***
## year2 0.045083 0.008613 5.23 1.66e-07 ***
## week 0.009038 0.001000 9.04 < 2e-16 ***
## avgtemp.s -0.006311 0.003367 -1.87 0.0609 .
## Age.s 0.010812 0.007633 1.42 0.1566
## chcem_alley_d1.s 0.013666 0.005714 2.39 0.0168 *
## handtotalttb_d1.s -0.001926 0.004168 -0.46 0.6441
## se_exit_d1.s -0.001069 0.004583 -0.23 0.8156
## se_squeeze_d1.s -0.003165 0.004691 -0.67 0.4999
## sftlat_supp.s -0.005761 0.005034 -1.14 0.2525
## nsftlat_supp.s -0.004737 0.006143 -0.77 0.4406
## ---
## Signif. codes: 0 '***' 0.001 '**' 0.01 '*' 0.05 '.' 0.1 ' ' 1
##
## Dispersion model:
## Estimate Std. Error z value Pr(>|z|)
## (Intercept) -2.56495 0.04081 -62.85 < 2e-16 ***
## year2 0.32741 0.05393 6.07 1.27e-09 ***
## ---
## Signif. codes: 0 '***' 0.001 '**' 0.01 '*' 0.05 '.' 0.1 ' ' 1

res.elev = simulateResiduals(elev, n=2000)
plotQQunif(res.elev)


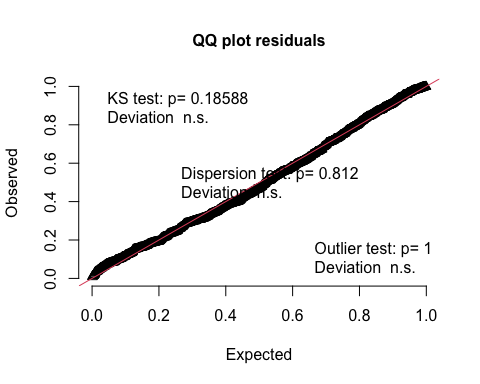


resid.elev <- resid(elev)
pred.elev <- predict(elev)
hist(resid.elev)


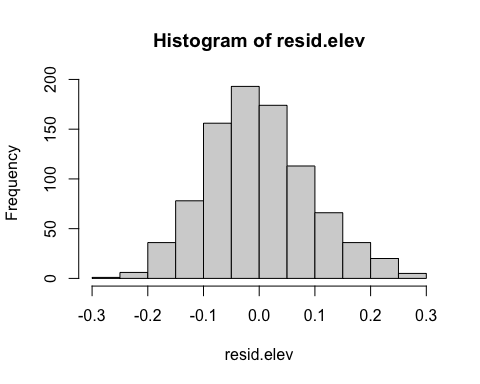


plot(pred.elev, resid.elev)


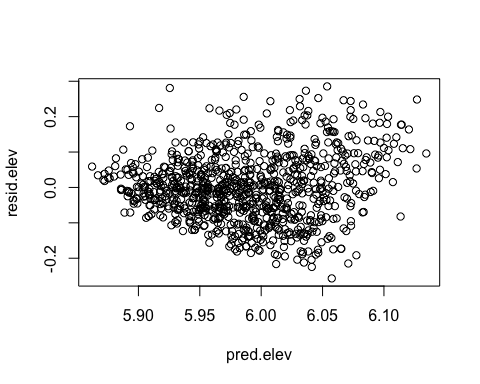


#tab_model(elev, show.se = TRUE, show.ci = FALSE, show.stat = TRUE, digits = 3, digits.p = 3)

pred.elev <- ggpredict(elev, terms = c("chcem_alley_d1.s"))

## Model has log-transformed response. Back-transforming predictions to
## original response scale. Standard errors are still on the transformed
## scale.

(elev <- plot(pred.elev, rawdata = TRUE) +
 labs(title = "Predicted Relationship for Elevation (m)",
 x = "Standardized Chute Duration (s)",
 y = "Average Elevation (m)"))

## Warning: Argument `rawdata` is deprecated and will be removed in the future.
## Please use `show_data` instead.

## Data points may overlap. Use the `jitter` argument to add some amount of
## random variation to the location of data points and avoid overplotting.


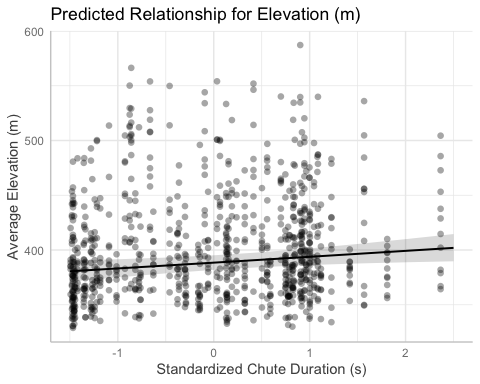


### Supplement

supp <- glmmTMB(mean_supp ~ year + week + week^2 + avgtemp.s + Age.s + chcem_alley_d1.s + handtotalttb_d1.s + se_exit_d1.s + se_squeeze_d1.s + sftlat_supp.s + nsftlat_supp.s + (1|Subjects), data = dat.week, dispformula = ~year) #log does not improve model fit, adding year improved heteroskedasticity
summary(supp)

## Family: gaussian ( identity )
## Formula:
## mean_supp ~ year + week + week^2 + avgtemp.s + Age.s + chcem_alley_d1.s +
## handtotalttb_d1.s + se_exit_d1.s + se_squeeze_d1.s + sftlat_supp.s +
## nsftlat_supp.s + (1 | Subjects)
## Dispersion: ~year
## Data: dat.week
##
## AIC BIC logLik deviance df.resid
## 11641.1 11708.1 -5806.5 11613.1 870
##
## Random effects:
##
## Conditional model:
## Groups Name Variance Std.Dev.
## Subjects (Intercept) 3507 59.22
## Residual NA NA
## Number of obs: 884, groups: Subjects, 49
##
## Conditional model:
## Estimate Std. Error z value Pr(>|z|)
## (Intercept) 1070.9484 54.9811 19.478 < 2e-16 ***
## year2 -150.9472 14.7284 -10.249 < 2e-16 ***
## week -14.1781 1.8064 -7.849 4.21e-15 ***
## avgtemp.s 2.0059 6.0735 0.330 0.7412
## Age.s -19.1091 10.8531 -1.761 0.0783 .
## chcem_alley_d1.s -20.0592 9.2019 -2.180 0.0293 *
## handtotalttb_d1.s 2.9824 7.0489 0.423 0.6722
## se_exit_d1.s 0.2582 7.6346 0.034 0.9730
## se_squeeze_d1.s 8.8878 7.9853 1.113 0.2657
## sftlat_supp.s 4.6187 8.2289 0.561 0.5746
## nsftlat_supp.s 7.2753 9.3691 0.777 0.4374
## ---
## Signif. codes: 0 '***' 0.001 '**' 0.01 '*' 0.05 '.' 0.1 ' ' 1
##
## Dispersion model:
## Estimate Std. Error z value Pr(>|z|)
## (Intercept) 4.98935 0.03987 125.15 < 2e-16 ***
## year2 0.20888 0.05201 4.02 5.92e-05 ***
## ---
## Signif. codes: 0 '***' 0.001 '**' 0.01 '*' 0.05 '.' 0.1 ' ' 1

res.supp = simulateResiduals(supp, n = 2000)
plotQQunif(res.supp)


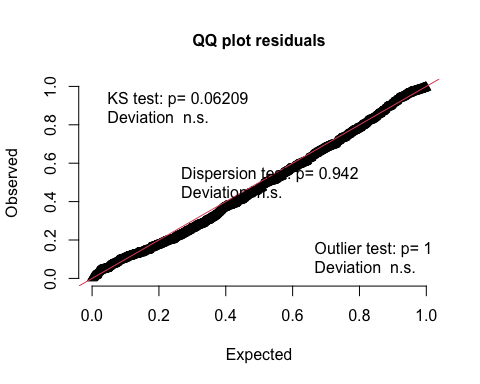


resid.supp <- resid(supp)
pred.supp <- predict(supp)

hist(resid.supp)


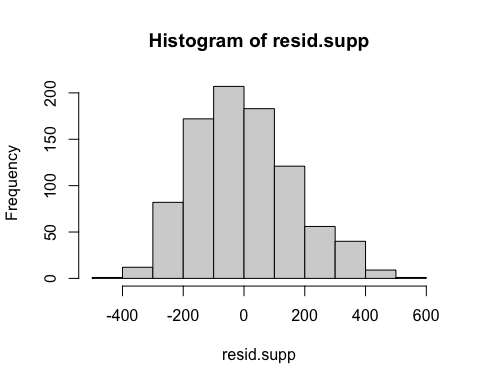


plot(pred.supp, resid.supp)


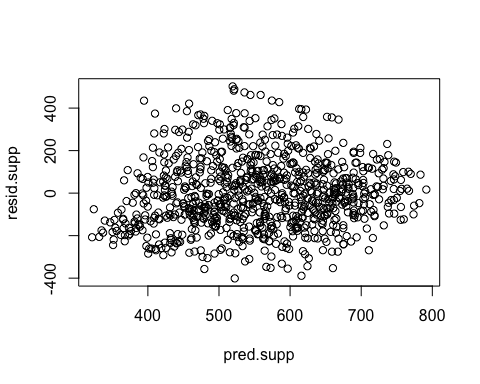


#tab_model(supp, show.se = TRUE, show.ci = FALSE, show.stat = TRUE, digits = 3, digits.p = 3)

### Water

water <- glmmTMB(log(mean_water) ~ year + week + week^2 + avgtemp.s + Age.s + chcem_alley_d1.s + handtotalttb_d1.s + se_exit_d1.s + se_squeeze_d1.s + sftlat_supp.s + nsftlat_supp.s + (1|Subjects), data = dat.week)
summary(water)

## Family: gaussian ( identity )
## Formula: log(mean_water) ~ year + week + week^2 + avgtemp.s + Age.s +
## chcem_alley_d1.s + handtotalttb_d1.s + se_exit_d1.s + se_squeeze_d1.s +
## sftlat_supp.s + nsftlat_supp.s + (1 | Subjects)
## Data: dat.week
##
## AIC BIC logLik deviance df.resid
## 54.4 116.6 -14.2 28.4 871
##
## Random effects:
##
## Conditional model:
## Groups Name Variance Std.Dev.
## Subjects (Intercept) 0.01525 0.1235
## Residual 0.05484 0.2342
## Number of obs: 884, groups: Subjects, 49
##
## Dispersion estimate for gaussian family (sigma^2): 0.0548
##
## Conditional model:
## Estimate Std. Error z value Pr(>|z|)
## (Intercept) 4.843122 0.080098 60.46 < 2e-16 ***
## year2 -0.070093 0.022464 -3.12 0.00181 **
## week 0.031025 0.002589 11.98 < 2e-16 ***
## avgtemp.s -0.065865 0.008594 -7.66 1.81e-14 ***
## Age.s 0.029421 0.020443 1.44 0.15010
## chcem_alley_d1.s 0.029888 0.014770 2.02 0.04301 *
## handtotalttb_d1.s -0.001895 0.011100 -0.17 0.86445
## se_exit_d1.s -0.017319 0.011489 -1.51 0.13170
## se_squeeze_d1.s 0.001733 0.012458 0.14 0.88937
## sftlat_supp.s -0.007139 0.013061 -0.55 0.58464
## nsftlat_supp.s -0.007011 0.016206 -0.43 0.66531
## ---
## Signif. codes: 0 '***' 0.001 '**' 0.01 '*' 0.05 '.' 0.1 ' ' 1

res.water = simulateResiduals(water, n = 2000)
plotQQunif(res.water)


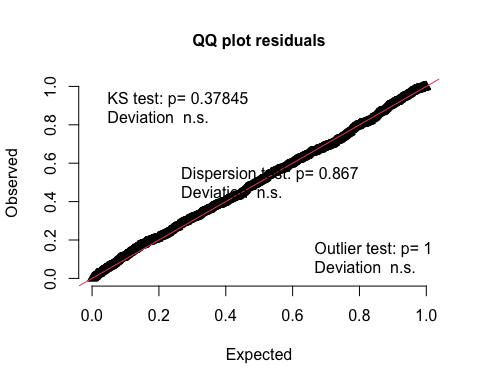


resid.water <- resid(water)
pred.water <- predict(water)

hist(resid.water)


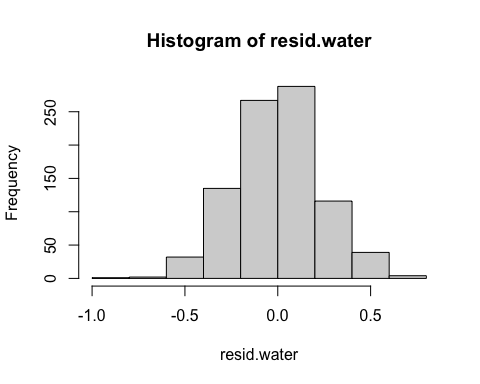


plot(pred.water, resid.water)


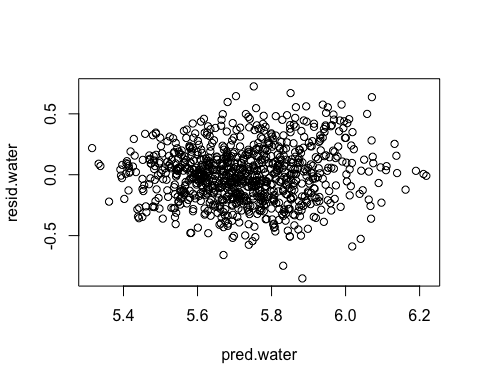


#tab_model(water, show.se = TRUE, show.ci = FALSE, show.stat = TRUE, digits = 3, digits.p = 3)

### Loaf

loaf <- glmmTMB(mean_loaf ~ year + week + week^2 + avgtemp.s + Age.s + chcem_alley_d1.s + handtotalttb_d1.s + se_exit_d1.s + se_squeeze_d1.s + sftlat_supp.s + nsftlat_supp.s + (1|Subjects), data = dat.week) #log not necessary to improve model fit
summary(loaf)

## Family: gaussian ( identity )
## Formula:
## mean_loaf ~ year + week + week^2 + avgtemp.s + Age.s + chcem_alley_d1.s +
## handtotalttb_d1.s + se_exit_d1.s + se_squeeze_d1.s + sftlat_supp.s +
## nsftlat_supp.s + (1 | Subjects)
## Data: dat.week
##
## AIC BIC logLik deviance df.resid
## 8819.3 8881.5 -4396.7 8793.3 871
##
## Random effects:
##
## Conditional model:
## Groups Name Variance Std.Dev.
## Subjects (Intercept) 62.27 7.891
## Residual 1179.46 34.343
## Number of obs: 884, groups: Subjects, 49
##
## Dispersion estimate for gaussian family (sigma^2): 1.18e+03
##
## Conditional model:
## Estimate Std. Error z value Pr(>|z|)
## (Intercept) 130.267066 11.451574 11.375 <2e-16 ***
## year2 -5.799799 2.931103 -1.979 0.0478 *
## week 0.004712 0.379090 0.012 0.9901
## avgtemp.s 0.059694 1.259139 0.047 0.9622
## Age.s -3.958132 1.727382 -2.291 0.0219 *
## chcem_alley_d1.s 0.016262 1.622638 0.010 0.9920
## handtotalttb_d1.s -0.798095 1.385285 -0.576 0.5645
## se_exit_d1.s -1.002402 1.418174 -0.707 0.4797
## se_squeeze_d1.s -2.691353 1.514383 -1.777 0.0755 .
## sftlat_supp.s 0.100575 1.554005 0.065 0.9484
## nsftlat_supp.s -1.540860 1.600096 -0.963 0.3356
## ---
## Signif. codes: 0 '***' 0.001 '**' 0.01 '*' 0.05 '.' 0.1 ' ' 1

res.loaf = simulateResiduals(loaf, n = 2000)
plotQQunif(res.loaf) #outlier detected, but leaving in because data processing already occurred


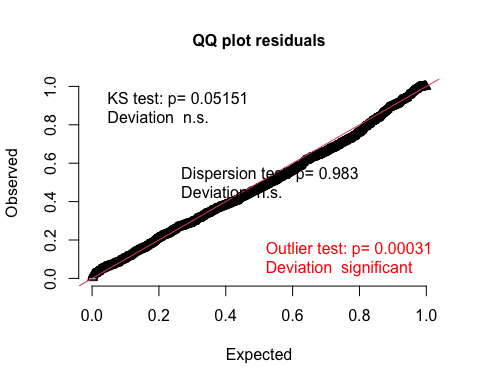


resid.loaf <- resid(loaf)
pred.loaf <- predict(loaf)
hist(resid.loaf)


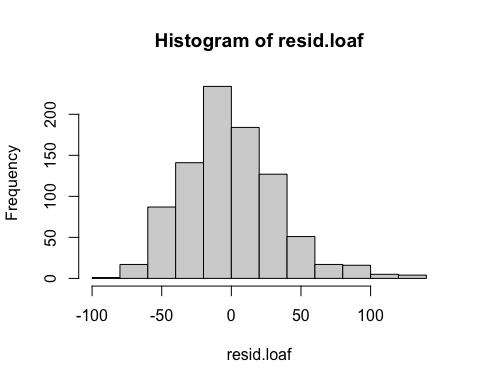


plot(pred.loaf, resid.loaf)


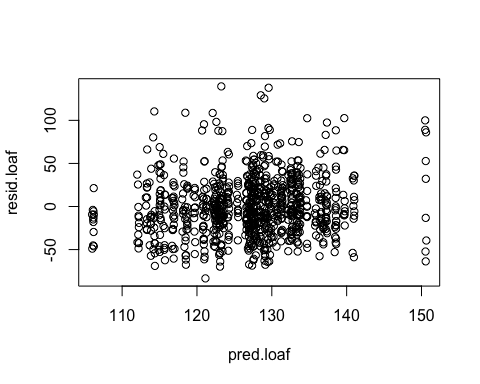


#tab_model(loaf, show.se = TRUE, show.ci = FALSE, show.stat = TRUE, digits = 3, digits.p = 3)

### AKDE 50% home range

akde50 <- glmmTMB(homerange.50 ~ Age.s + chcem_alley_d1.s + handtotalttb_d1.s + se_exit_d1.s + se_squeeze_d1.s + sftlat_supp.s + nsftlat_supp.s + year + (1|Subjects), data = akde.sn.yr)
summary(akde50)

## Family: gaussian ( identity )
## Formula:
## homerange.50 ~ Age.s + chcem_alley_d1.s + handtotalttb_d1.s +
## se_exit_d1.s + se_squeeze_d1.s + sftlat_supp.s + nsftlat_supp.s +
## year + (1 | Subjects)
## Data: akde.sn.yr
##
## AIC BIC logLik deviance df.resid
## 605.9 631.8 -292.0 583.9 67
##
## Random effects:
##
## Conditional model:
## Groups Name Variance Std.Dev.
## Subjects (Intercept) 8.026e-07 8.959e-04
## Residual 1.044e+02 1.022e+01
## Number of obs: 78, groups: Subjects, 49
##
## Dispersion estimate for gaussian family (sigma^2): 104
##
## Conditional model:
## Estimate Std. Error z value Pr(>|z|)
## (Intercept) 73.2132 4.3683 16.760 <2e-16 ***
## Age.s -0.2782 1.2354 -0.225 0.8219
## chcem_alley_d1.s 2.3489 1.2811 1.834 0.0667 .
## handtotalttb_d1.s -0.5552 1.1061 -0.502 0.6157
## se_exit_d1.s 0.8702 1.1913 0.730 0.4651
## se_squeeze_d1.s -1.9245 1.2274 -1.568 0.1169
## sftlat_supp.s -0.1371 1.2572 -0.109 0.9132
## nsftlat_supp.s 1.3611 1.2284 1.108 0.2678
## year -4.4015 2.6508 -1.660 0.0968 .
## ---
## Signif. codes: 0 '***' 0.001 '**' 0.01 '*' 0.05 '.' 0.1 ' ' 1

res.50 = simulateResiduals(akde50, n = 2000)
plot(res.50) #in this case using DHARMa because no longer large sample size, since one value per season


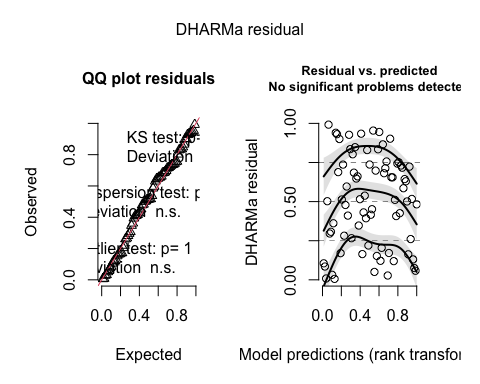


#tab_model(akde50, show.se = TRUE, show.ci = FALSE, show.stat = TRUE, digits = 3, digits.p = 3)

pred.akde <- ggpredict(akde50, terms = c("chcem_alley_d1.s"))
(akde <- plot(pred.akde, rawdata = TRUE) +
 labs(title = "Predicted Relationship for Core Home Range (ha)",
 x = "Standardized Chute Duration",
 y = "Core Home Range (ha)"))

## Warning: Argument `rawdata` is deprecated and will be removed in the future.
## Please use `show_data` instead.

## Data points may overlap. Use the `jitter` argument to add some amount of
## random variation to the location of data points and avoid overplotting.


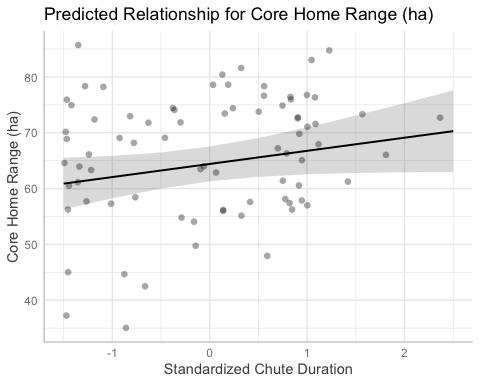


### SN degree strength model

sn <- glmmTMB(strength ~ year + Age.s + chcem_alley_d1.s + handtotalttb_d1.s + se_exit_d1.s + se_squeeze_d1.s+ sftlat_supp.s + nsftlat_supp.s+ (1|Subjects), data = akde.sn.yr)
summary(sn)

## Family: gaussian ( identity )
## Formula:
## strength ~ year + Age.s + chcem_alley_d1.s + handtotalttb_d1.s +
## se_exit_d1.s + se_squeeze_d1.s + sftlat_supp.s + nsftlat_supp.s +
## (1 | Subjects)
## Data: akde.sn.yr
##
## AIC BIC logLik deviance df.resid
## 7.4 33.3 7.3 -14.6 67
##
## Random effects:
##
## Conditional model:
## Groups Name Variance Std.Dev.
## Subjects (Intercept) 0.009346 0.09667
## Residual 0.039872 0.19968
## Number of obs: 78, groups: Subjects, 49
##
## Dispersion estimate for gaussian family (sigma^2): 0.0399
##
## Conditional model:
## Estimate Std. Error z value Pr(>|z|)
## (Intercept) 1.591230 0.091031 17.480 <2e-16 ***
## year -0.105716 0.054263 -1.948 0.0514 .
## Age.s -0.025321 0.028575 -0.886 0.3756
## chcem_alley_d1.s 0.005793 0.029009 0.200 0.8417
## handtotalttb_d1.s -0.013305 0.024099 -0.552 0.5809
## se_exit_d1.s 0.005505 0.025957 0.212 0.8320
## se_squeeze_d1.s -0.045787 0.027402 -1.671 0.0947 .
## sftlat_supp.s -0.012179 0.027064 -0.450 0.6527
## nsftlat_supp.s 0.040488 0.027439 1.476 0.1401
## ---
## Signif. codes: 0 '***' 0.001 '**' 0.01 '*' 0.05 '.' 0.1 ' ' 1

res.sn = simulateResiduals(sn, n = 2000)
plot(res.sn) #in this case using DHARMa because no longer large sample size, since one value per season


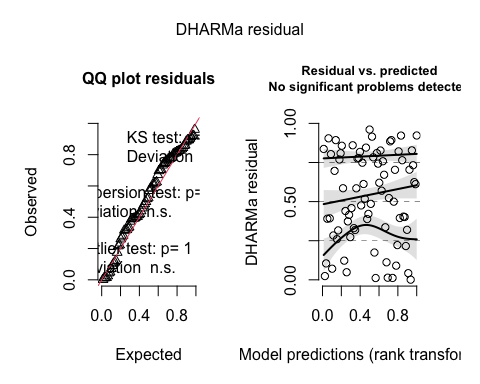


#tab_model(sn, show.se = TRUE, show.ci = FALSE, show.stat = TRUE, digits = 3, digits.p = 3)

## check vifs

mod <- lmer(mean_elev ~ year + week + avgtemp.s + Age.s + handtotalttb_d1.s + chcem_alley_d1.s + se_exit_d1.s + se_squeeze_d1.s + sftlat_supp.s + nsftlat_supp.s + (1|Subjects), data = dat.week)
car::vif(mod) #all around one

## year week avgtemp.s Age.s
## 1.604919 1.170297 1.202476 1.034582
## handtotalttb_d1.s chcem_alley_d1.s se_exit_d1.s se_squeeze_d1.s
## 1.170952 1.209019 1.214775 1.268499
## sftlat_supp.s nsftlat_supp.s
## 1.237217 1.109132
